# Successful endoscopic biliary intervention for duodenal peridiverticular papilla using a novel traction device

**DOI:** 10.1055/a-2351-3000

**Published:** 2024-07-08

**Authors:** Kyosuke Tanaka, Junya Tsuboi, Kento Naruse, Hiroki Yukimoto, Shunsuke Nojiri, Tomoyuki Nomura

**Affiliations:** 1 157580 Department of Internal Medicine, Inabe General Hospital, Inabe, Japan; 2 157580 Department of Gastroenterology, Inabe General Hospital, Inabe, Japan; 3 Department of Gastroenterology, Mie University Graduate School of Medicine, Tsu, Japan; 4 Department of Gastroenterology and Metabolism, Nagoya City University Graduate School of Medical Sciences, Nagoya, Japan

Periampullary diverticulum is defined as herniation of the mucosa or submucosa through a defect in the muscular layer around the papilla 1 . Achieving selective biliary cannulation for peridiverticular papilla is challenging 1 , and several endoscopic devices and techniques have recently been developed 1 2 3 4 . Herein, we present a case of successful endoscopic biliary intervention using a novel traction device for the peridiverticular papilla in a patient with choledocholithiasis.

A 92-year-old woman was diagnosed with choledocholithiasis on computed tomography after treatment for aspiration pneumonia. Endoscopic sphincterotomy and stone extraction were performed ( Video 1 ). On endoscopy, a duodenal papilla was identified as being situated within the diverticulum at its periphery ( Fig. 1 ); however, biliary cannulation failed because of poor visualization of the papillary orifice. Therefore, we used a novel traction device (EndoTrac; TOP Corporation, Tokyo, Japan) comprising a line with a clinch-knotted loop encased in a plastic sheath 5 . An endoclip was inserted through the endoscopic channel to the tip and the EndoTrac line was tied to it. The endoscope and traction device were simultaneously inserted into the duodenum. The endoclip with EndoTrac was positioned at the distal mucosa of the diverticular edge. The tip of the plastic sheath was advanced toward the endoclip, and the EndoTrac was pushed for repositioning of the papilla. The duodenal papilla was moved from the diverticulum by manipulating the device ( Fig. 2 ). The traction force exerted by the device was adjusted to ensure optimal positioning of the duodenal papilla. The selective biliary cannulation was successful ( Fig. 3 ). Sphincterotomy and balloon dilatation were performed ( Fig. 4 ), and stone extraction was completed ( Fig. 5 ). Endoscopic biliary procedures using the EndoTrac device can provide good visualization of the papilla and facilitate biliary cannulation for the peridiverticular papilla.

Successful biliary cannulation for a duodenal peridiverticular papilla using a novel traction device (EndoTrac).Video 1

**Fig. 1:**
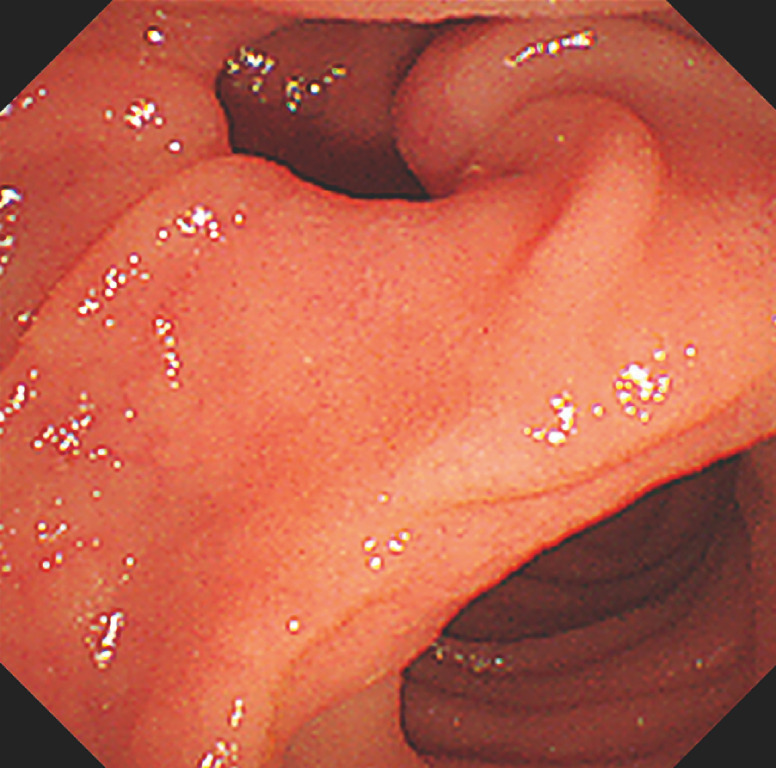
Endoscopic visualization of the peridiverticular papilla in the duodenum.

**Fig. 2:**
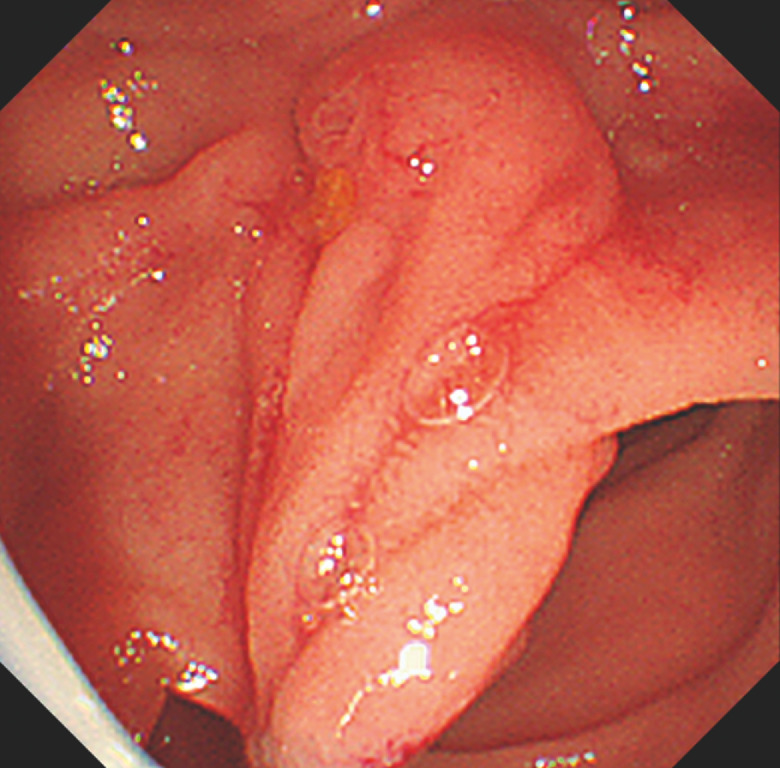
When the traction device is manipulated, the duodenal papilla is moved out of the diverticulum.

**Fig. 3:**
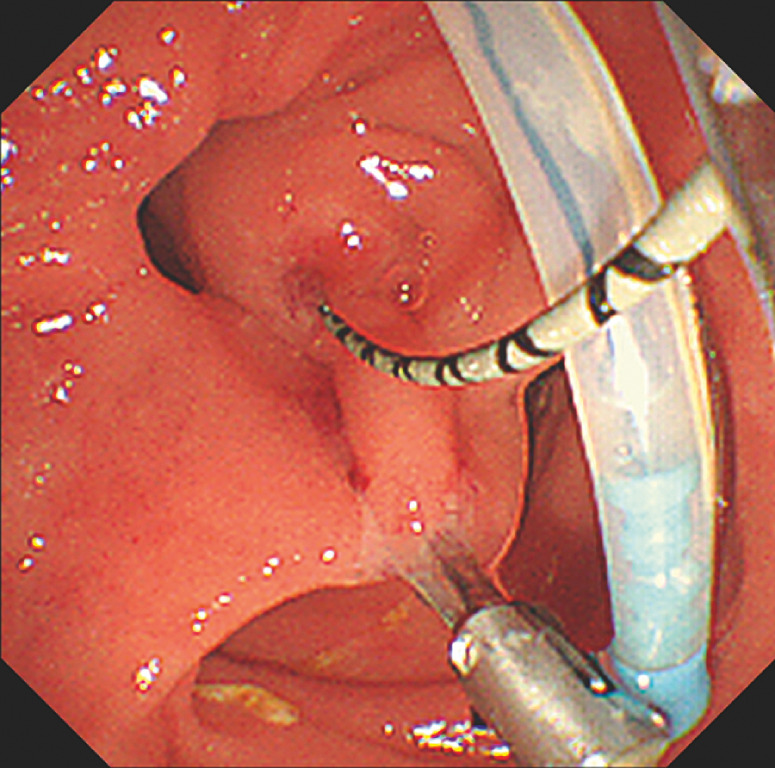
Successful selective biliary cannulation after repositioning of the papilla.

**Fig. 4:**
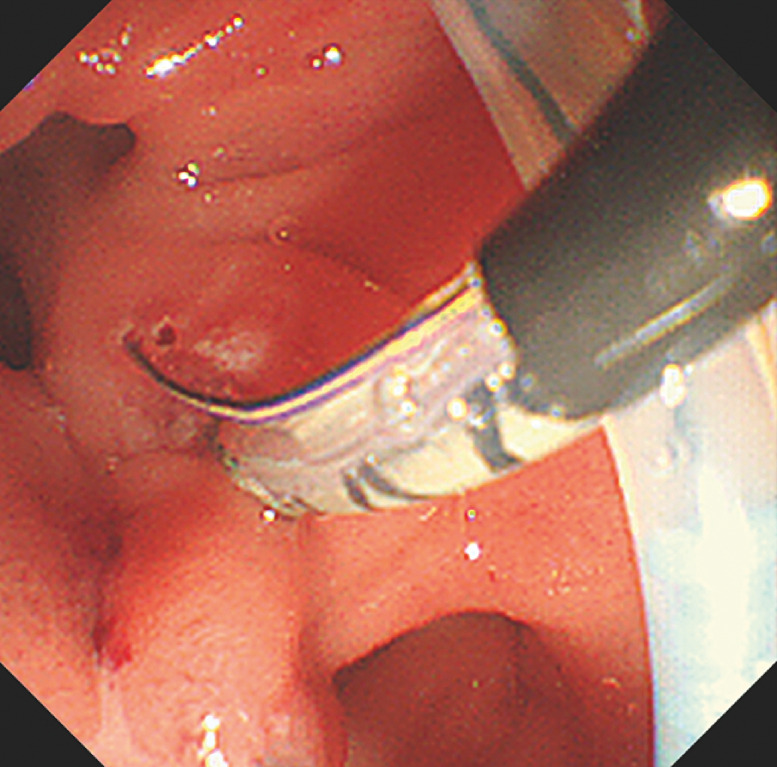
Endoscopic sphincterotomy is performed.

**Fig. 5:**
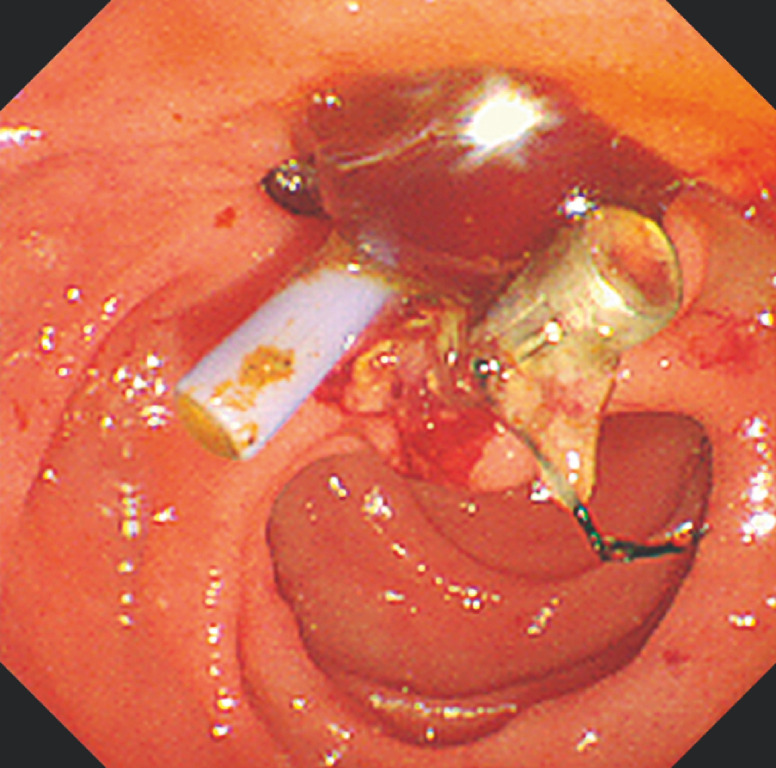
After stone extraction and placement of a biliary drainage tube, the line used for traction is cut using an endocutter.

Endoscopy_UCTN_Code_TTT_1AR_2AC

## Notes

Endoscopy E-Videos https://eref.thieme.de/e-videos
*E-Videos* is an open access online section of the journal *Endoscopy* , reporting on interesting cases and new techniques in gastroenterological endoscopy. All papers include a high-quality video and are published with a Creative Commons CC-BY license. Endoscopy E-Videos qualify for HINARI discounts and waivers and eligibility is automatically checked during the submission process. We grant 100% waivers to articles whose corresponding authors are based in Group A countries and 50% waivers to those who are based in Group B countries as classified by Research4Life (see: https://www.research4life.org/access/eligibility/ ). This section has its own submission website at https://mc.manuscriptcentral.com/e-videos .

## References

[1] Altonbary AY Bahgat MH Endoscopic retrograde cholangiopancreatography in periampullary diverticulum: the challenge of cannulation World J Gastrointest Endosc 2016 8 282 287 10.4253/wjge.v8.i6.282 27014423 PMC4804185

[2] Inoue R Kawakami H Kubota Y Endoscopic biliary intervention using traction devices for periampullary diverticulum Intern Med 2019 58 2797 2801 10.2169/internalmedicine.2804-19 31178511 PMC6815890

[3] Ishii T Kin T Katanuma A Successful intervention using multiloop traction for cases with difficult biliary cannulation due to periampullary diverticula Dig Endosc 2021 33 e111 e113 10.1111/den.14003 34046941

[4] Iwano K Toyonaga H Kin T Multiloop traction method during endoscopic hemostasis for post-sphincterotomy bleeding of the peridiverticular papilla Endoscopy 2022 54 E769 E770 35395687 10.1055/a-1795-7092 PMC9735241

[5] Tanaka S Toyonaga T Kaku H A novel traction device (EndoTrac) for use during endoscopic submucosal dissection Endoscopy 2019 51 E90 E91 30731488 10.1055/a-0830-4556

